# US measles outbreak: causes, consequences and the path forward

**DOI:** 10.1016/j.eclinm.2025.103174

**Published:** 2025-03-24

**Authors:** eClinicalMedicine

In 2000 the USA achieved measles elimination status. Since then, it has come very close to losing that status in 2019 and now, in 2025, it is facing a large, growing measles outbreak that has led to two fatalities, for the first time since 2015. The US Centers for Disease Control and Prevention (CDC) has reported over 222 cases so far in the latest outbreak, the majority of which have been in Texas with additional cases in Alaska, California, Georgia, Kentucky, New Jersey, New Mexico, New York City, and Rhode Island.

Measles has been on a rise in recent times. The measles virus is known to be one of the most contagious viruses known to humans, with a basic reproduction number (R0) of 12–18, and is completely preventable by vaccination since 1963. Despite this, in 2024, according to WHO, there were globally 151,103 cases of laboratory confirmed measles. At present, measles outbreaks are widespread and global: Pakistan (7148 cases), Thailand (6852), India (6203), Yemen (5000), and Ethiopia (4724) are all facing outbreaks. In January 2024, the UK Health and Security Agency declared a national incident, due to the rising number of measles cases in the country, despite regaining measles elimination status in 2021. This led to a total of 2911 laboratory confirmed measles cases in England in 2024, the highest number of cases recorded annually since 2012.

It is not surprising that measles outbreaks are a more frequent occurrence now. The CDC reports that global measles activity is increasing and vaccination coverage in the USA in on the decline. During the 2023–24 school year, vaccination coverage among kindergartners in the USA decreased for all reported vaccines from the year before, ranging from 92.7% (in 2022–23) to 92.3% for diphtheria, tetanus, and acellular pertussis vaccine (DTaP) and from 93% (in 2022–23) to 92.7% for measles, mumps, and rubella vaccine (MMR). Additionally, exemptions from one or more vaccines among kindergartners increased to 3.3% from 3.0% in the year before.

For herd immunity to come into action, according to WHO targets, at least 95% of a population needs to be vaccinated. In Gaines County in western Texas, where the current US measles outbreak began, Texas Department of State Health services (DSHS) reports that as few as 82% of kindergartners are vaccinated against the virus. The current Texas outbreak has predominantly affected children, with 153 of the 198 cases occurring in individuals younger than 18 years. The DSHS also reports that 80 of the confirmed cases were in individuals who had not received the MMR vaccine, whereas 113 cases had unknown vaccination status. The one fatality in Texas, was an unvaccinated child. The second death was of an unvaccinated adult from New Mexico.

Vaccine coverage is reducing globally. In the UK, among the 2911 cases in 2024, the majority (1722 of 2911, 59.2%) were in children aged 10 years or younger. National Health Service (NHS) data shows that, during 2023–24 in England, vaccination coverage decreased across all of the 14 coverage measures reported. Additionally, none of the vaccines met the 95% population target and MMR1 coverage at age 5 years decreased to 91.9%, the lowest level since 2010–11.

In addition to disruption of immunisation programmes and campaigns due to the COVID-19 pandemic, vaccine hesitancy and scepticism among families and communities, have contributed to declining vaccine coverage. The increased resurgence of the anti-vaccine movement in recent years has not helped with spread of rampant misinformation, especially via social media and unverified news coverage. Vaccine hesitancy can arise from a variety of reasons, some of which can be associated with a specific vaccine such as the now debunked and disproved yet persistent belief that the MMR vaccine can cause autism or the inconclusive link between hepatitis B vaccine and multiple sclerosis. Other causes of vaccine hesitancy can include fear of needles or side-effects, concerns related to vaccine ingredients (eg, thimerosal), or apprehensions associated with newly developed vaccines, such as for COVID-19.

President Trump's second term has brought uncertainty in the future of US and global health landscape. His actions including withdrawing from WHO, cutting funds for the National Institutes of Health and United States Agency for International Development are worrisome and the future of vaccines and vaccine uptake are not shielded from this. The newly appointed US Department of Health and Human service secretary, Robert J Kennedy Jr is openly known to be anti-vaccine and the founder of the nonprofit Children's Health Defense, the most well funded anti-vaccine organisation in the USA. It is the early days of Trump 2.0 and already we have seen Vaccine Advisory Meetings cancelled and postponed indefinitely, CDC promotional vaccine campaigns halted and plans to review the childhood vaccination schedule. In response to the current measles outbreak, Kennedy has taken a U-turn on his earlier stance and said “Vaccines not only protect individual children from measles, but also contribute to community immunity.” However, he did also continue to say that vaccination is a personal choice and heavily pushed unproven theories that vitamin A, cod liver oil, and steroids are effective measles treatments.

Vaccination is the only known prevention for measles, and there is no specific treatment to date. Governments around the world are running campaigns urging parents to make sure their children receive their due vaccinations. Despite these efforts, the rise in measles cases, an altogether preventable disease, is disheartening. The recent outbreaks should be taken as a wakeup call to reignite our efforts towards global public health and vaccination efforts. People should be encouraged to listen to factual scientific evidence and verified sources. Awareness on the safety of the measles vaccine needs to be spread to combat the viral misinformation against it. Targeted campaigns conducted under global collaboration with agencies such as WHO and UNICEF involved, are necessary to ensure all children are vaccinated, in high-income, middle-income, and low-income countries alike. Leaving a better world for our next generation includes making the next generation healthy enough to enjoy it.

